# Microglia/macrophage diversities in central nervous system physiology and pathology

**DOI:** 10.1111/cns.13257

**Published:** 2019-12-02

**Authors:** Di Xie, Maxine He, Xiaoming Hu

**Affiliations:** ^1^ Pittsburgh Institute of Brain Disorders and Recovery Department of Neurology University of Pittsburgh School of Medicine Pittsburgh PA USA; ^2^ Neuroscience Program University of Illinois at Urbana‐Champaign Champaign IL USA

Since the discovery of microglia 100 years ago, the functions of this glial cell population in the central nervous system (CNS) have been widely investigated. Microglia were initially recognized as the phagocytic cells in the CNS to clear abnormal cells or invading pathogens.^1^ The later advent of cell‐specific transgenic mice and two‐photon imaging systems allows real‐time in vivo imaging of microglia, revealing highly dynamic behaviors of these cells. The so‐called “resting” microglia in the normal CNS do not remain stationary. Instead, they actively survey their extracellular space and cellular neighborhood using their highly mobile processes and thus play essential roles in host perception of internal or external stimulations.^2^ When activated, microglia experience morphological transformation and launch defense responses to clear damaged cells and fight against abnormalities. Microglia also release cytokines, chemokines, trophic factors, and other immune modulators to modify the CNS microenvironment and influence functions of neighboring cells. It is becoming increasingly clear now that microglia actively interact with other CNS components, maintain CNS integrity, and regulate brain functions.^2^, ^3^

In addition to microglia within the brain parenchyma, there are some other myeloid cells within the CNS, which are referred to as border‐associated macrophages due to their special locations at the CNS “borders” in the perivascular spaces, the leptomeningeal spaces, and the choroid plexus. These macrophages actively interact with the vasculature, playing critical roles as immune sentinels, scavengers, and function modulators.^2^

Despite the consensus view about the importance of microglia and macrophages in the CNS under physiological conditions, their functions in a diseased or injured brain remain controversial for a long time. Some studies documented the destructive role of microglia/macrophages in brain pathologies as highly activated microglia release a plethora of neurotoxic factors, including inflammatory cytokines, chemokines, and free radicals. In support of this view, microglia depletion has been reported to result in neuroprotection in experimental models of hemorrhagic stroke,^4^ chronic cerebral hypoperfusion,^5^ traumatic brain injury (TBI),^6^ and Alzheimer's disease (AD).^7^ In contrast, quite a few studies documented that the removal of microglia enhanced neuroinflammation and thus exacerbated the neurological deficits after brain injuries or neurodegenerations, suggesting beneficial roles of microglia in the presence of CNS pathologies.^8^, ^9^, ^10^, ^11^ In an effort to elucidate the apparent divergence in perspectives of microglia functions, neuroscientists extrapolated the concept of immune cell polarization in the peripheral immune system and investigated the diversity of microglia phenotypes in CNS disorders. Accumulating evidence supports that microglia do not constitute uniformed cell populations in the compromised CNS. Instead, they polarize into a variety of phenotypes at different stages of injuries or diseases. These phenotypes may have distinct roles. In particular, the classically activated or proinflammatory phenotype is characterized by the release of proinflammatory factors and free radicals that impair CNS integrity. By contrast, the alternatively activated or antiinflammatory phenotype possesses functions or expresses proteins that preserve brain tissue or improve CNS repair.^12^, ^13^ Such dichotomic definition of microglia phenotype was later superseded by a view of a broad spectrum of interchangeable functional states in the lesioned nervous system. More and more microglia subpopulations with expressions of a panel of unique signature genes have been identified in different disease models. For example, Arginase 1 (Arg1)^+^ microglia in response to prolonged interleukin (IL)‐1β production have been reported to reduce Aβ plaque deposition in an animal model of AD.^14^ The tumor necrosis factor‐α (TNF‐α)–producing microglia in hippocampal impaired working memory under acute stress.^15^ Recent development in single‐cell technology allows the discovery of more microglia subpopulations. A unique CD11c^+^ microglia subtype has been identified as disease‐associated microglia (DAM) in the aged brains and AD brains.^16^ A cluster of Apoe^+^Ccl5^+^ microglia has been observed at the onset of recovery from nerve injury.^17^ A recent study showed that CNS‐resident macrophages also quickly transformed into context‐dependent subsets during brain inflammation.^18^ In addition, bone marrow‐derived macrophages that infiltrate into the brain in case of blood‐brain barrier breach bring in more subsets of myeloid cells.^19^ The functional significance of these microglia/macrophage subpopulations awaits further elucidation.

Adding extra layers of complexity, there are a variety of factors, including age, sex, and environmental cues that increase the diversities of microglia/macrophages. The lack of preclinical studies in aged animals has resulted in failures of neuroprotective strategies in clinical trials.^20^, ^21^ Age‐related changes in microglia have been well‐accepted.^22^ Increased microglial activation in the aged brain could be visualized using positron emission tomography (PET).^23^, ^24^ Morphologically, aged microglia display increment in soma volume and shortening in processes. Consequently, the survey territory of individual microglia decreases. To compensate for the decrease in process coverage, aged microglia proliferate and cluster together, whereas their homogeneous spatial distribution is disturbed.^25^ Functionally, the clearance capacities of aged microglia decrease due to the overload of misfolded proteins or degraded cellular components.^26^ Additionally, microglia are primed by elevated inflammatory cues in the aged brain. Primed microglia are prone to respond to second inflammatory stimuli and generate hyperactive responses.^27^ However, some other in vitro and in vivo studies argued that senescent microglia showed reduced responses to noxious stimulations.^28^ Thorough transcriptome analysis and functional evaluation are required to elucidate alterations in senescent microglia and/or macrophages, and their contribution to normal aging and age‐related diseases.

Sex is another factor that impacts brain functions.^29^ It has long been noticed that the female and male microglia show differences in brain colonization in an area and time‐specific manner. For example, in the preoptic area, males have overall more microglia, especially more amoeboid microglia early in postnatal development. Such difference is hormone‐dependent as estradiol treatment to females at P0 and P1 increases microglial counts and numbers of amoeboid microglia to the male level. As in juveniles and adults, male and female microglia exhibit differences in cell number and morphology.^30^ Sexually dimorphism in microglial functions has also been reported. Male microglia exhibit higher mobility in response to chemoattractant^31^ and have a higher level of antigen‐presenting capacity compared with female microglia.^32^ Not only sex differences impact the functions and properties of microglia, but also microglia, in turn, participate in brain sexual differentiation. It was found that microglial activation is necessary to induce the masculine pattern of dendritic spines in the preoptic neurons and appropriate sexual behaviors in adults.^33^

Some other factors also contribute to microglia diversity. The influence of stress, alcohol consumption, and diet on microglial activity has been reported, implicating the impact of lifestyle on microglia.^34^, ^35^ In addition, environmental exposure impacts microglia phenotypes in many aspects. It was found that prenatal exposure to air pollution causes increased proinflammatory cytokine secretions by microglia.^36^ The elevated level of ozone also promotes the proinflammatory responses in microglia.^37^

There is an increasing recognition of microglia diversity and its importance in CNS homeostasis and pathologies. With the bloom of whole‐genome analysis in couple with transcriptomic and proteomic approaches, the heterogeneity of microglia/macrophage subpopulation is being further dissected. Many condition‐specific or disease‐specific microglia/macrophages have been defined while their functions remain elusive.^16^, ^17^ In addition, more and more extracellular factors and intracellular molecules that regulate phenotypic changes in phagocytes are identified.^12^ Selective manipulation of microglia/macrophage phenotypes has been shown to improve outcomes in different preclinical models of neurological disorders, including TBI, stroke, and Parkinson's disease^38^, ^39^, ^40^, ^41^, ^42^, ^43^ and may provide promising therapeutic strategies that can be translated into clinical use. This special issue includes a collection of original research papers and review articles that covers a topic regarding microglia/macrophage diversities, with an intention to provide updated views of microglia/macrophage phenotypic variety in response to CNS injuries and diseases, and the therapeutic potential of strategies that adjust microglia responses.

## CONFLICT OF INTEREST

None.
